# Iron Deficiency Anemia and Oxidative Stress in Type 2 Diabetic Patients on Metformin: A Meta-Analysis

**DOI:** 10.7759/cureus.84386

**Published:** 2025-05-19

**Authors:** Arooba Rubbani, Danial Khalid, Savera Nadeem, Ayesha Ghazal Jamali, Saiyeda Asma B Rizvi, Aftab Rabbani, Sundeep Sahitia, Malik Asfand Yar, Amro A. M. Albatrawi, Maha M Abdul-Latif

**Affiliations:** 1 Medicine, University Hospitals of Leicester NHS Trust, Leicester, GBR; 2 Medicine, University Hospitals Bristol and Weston NHS Trust, Bristol, GBR; 3 Medicine, Karachi Institute of Medical Sciences (KIMS) Combined Military Hospital (CMH), Karachi, PAK; 4 Medicine, Liaquat University of Medical and Health Sciences, Jamshoro, PAK; 5 Physiology, Dow International Medical College, Karachi, PAK; 6 Medicine, Sharif Medical City Hospital, Lahore, PAK; 7 Health and Hospital Management, Institute of Business Management, Karachi, PAK; 8 Acute Medicine, Queen Elizabeth Hospital, University Hospitals Birmingham (UHB) NHS, Birmingham, GBR; 9 Medicine, Cairo University, Cairo, EGY; 10 Ophthalmology, College of Medicine/Northern Border University, Arar, SAU

**Keywords:** antioxidant enzymes, iron deficiency anemia, metformin, oxidative stress, type 2 diabetes mellitus

## Abstract

Iron deficiency anemia (IDA), one of the most prevalent nutritional disorders globally, is increasingly recognized among patients with type 2 diabetes mellitus (T2DM). Metformin, the first-line antidiabetic drug, is widely used in T2DM management and known for its favorable metabolic effects. However, growing evidence suggests that long-term metformin use may contribute to micronutrient deficiencies, including vitamin B12 and potentially iron, which could impair hematological status and exacerbate oxidative stress in diabetic individuals. Oxidative stress, characterized by an imbalance between reactive oxygen species (ROS) and antioxidant defenses, plays a crucial role in the pathogenesis of diabetic complications. The interconnection between metformin-associated IDA and oxidative stress remains inadequately explored.

This meta-analysis aimed to assess the association between IDA and oxidative stress in patients with T2DM receiving metformin therapy. A systematic search was conducted in PubMed, Cochrane Central Register of Controlled Trials (CENTRAL), Scopus, ProQuest, and Google Scholar for studies published between January 2000 and March 2024. Studies were included if they reported both iron-related hematological indices and oxidative stress markers in adult patients with T2DM on metformin. Data were extracted and analyzed using the Comprehensive Meta-Analysis software, applying a random-effects model.

Three studies meeting the inclusion criteria were identified, encompassing 521 participants from Egypt, China, and Pakistan. Pooled analysis revealed significantly lower hemoglobin levels in metformin-treated patients with IDA (mean 11.6 ± 1.2 g/dL) compared to non-IDA controls (13.8 ± 1.4 g/dL; *P* < 0.001). Markers of oxidative stress were also significantly altered, with higher malondialdehyde (MDA: 3.9 ± 0.7 µmol/L vs. 2.4 ± 0.5 µmol/L) and lower antioxidant levels (superoxide dismutase [SOD], glutathione [GSH], and glutathione peroxidase [GPx]). Meta-regression indicated that higher HbA1c and longer diabetes duration were associated with worsened oxidative parameters. Sensitivity analysis confirmed the robustness of these findings.

In conclusion, IDA in patients with T2DM receiving metformin therapy is significantly associated with elevated oxidative stress and compromised antioxidant defenses. These findings underscore the importance of routine screening for anemia and oxidative stress markers in metformin-treated diabetic patients, while also emphasizing the need for further research to inform long-term therapeutic strategies.

## Introduction and background

Iron deficiency anemia (IDA), one of the most widespread nutritional deficiencies globally, affecting both developed and developing populations alike [1,2]. According to the Global Burden of Disease study covering 187 countries, IDA remains the leading cause of anemia, particularly among vulnerable groups such as preschool-aged children, pregnant women, and individuals with chronic conditions [3]. Notably, patients with type 2 diabetes mellitus (T2DM) appear to have a higher prevalence of IDA compared to non-diabetic individuals [4,5]. This comorbidity is clinically significant, as IDA may impair glycemic control, disrupt glucose homeostasis, and potentially increase the risk of diabetes-related complications [6]. Evidence suggests that addressing IDA can enhance metabolic stability and delay the onset of diabetic complications; however, iron supplementation itself must be approached with caution, as it has been shown to worsen glycemic outcomes in some patients with T2DM [7]. This necessitates an in-depth understanding of how IDA interacts with diabetic pathophysiology and treatment responses [8].

Metformin is the most widely prescribed first-line oral hypoglycemic agent for T2DM and is used by more than half of diabetic patients worldwide [9]. While it is known for its beneficial pleiotropic effects, including improved insulin sensitivity and endothelial function, its association with micronutrient deficiencies has raised concern. Although a meta-analysis of 31 studies found no significant link between metformin and IDA, it did report a strong association with vitamin B12 deficiency [10]. Some studies have found no difference in hemoglobin levels between metformin and non-metformin users [11], while others suggest that metformin may increase the risk of anemia, although often without specific classification [12]. Experimental studies offer additional mechanistic insights. Animal models have shown that metformin can improve certain hematological parameters in anemic states [13], while cell culture experiments suggest it may modulate intracellular iron levels [14]. These inconsistencies may be attributed to variations in study populations, treatment durations, and diagnostic criteria, leaving the relationship between metformin and IDA unresolved.

Iron plays a crucial role in oxygen transport and energy production, and its deficiency leads to symptoms such as fatigue, weakness, and reduced physical capacity-symptoms that are particularly problematic in patients with T2DM [15]. Moreover, iron deficiency is associated with pro-oxidative effects, which may exacerbate insulin resistance and other diabetes-related complications. Oxidative stress, a condition characterized by an imbalance between reactive oxygen species (ROS) production and antioxidant defenses, leads to cellular damage affecting lipids, proteins, and DNA [15] [16]. It is well-documented that oxidative stress is a key contributor to the pathogenesis of T2DM and its complications, including nephropathy, retinopathy, and cardiovascular diseases [17].

The role of metformin in this context is complex. Some hypotheses suggest that metformin may reduce vitamin B12 levels over time, impairing red blood cell synthesis and contributing to anemia [4]. Others point to metformin’s impact on the gastrointestinal tract and gut microbiota, which may interfere with the absorption of iron and other essential micronutrients [5]. Given the interrelated mechanisms of oxidative stress, inflammation, endothelial dysfunction, and nutrient malabsorption in T2DM, it is plausible that IDA may further amplify oxidative damage in these patients [6].

Despite the potential clinical implications, the association between IDA and oxidative stress in metformin-treated patients with T2DM remains underexplored. Therefore, this meta-analysis aims to assess and quantify the relationship between iron deficiency anemia and oxidative stress markers in individuals with T2DM taking metformin. Clarifying this relationship is essential for guiding screening strategies and optimizing long-term management in this high-risk population.

## Review

Methodology

Search Strategy

A systematic literature search was conducted across several electronic databases, including PubMed, Cochrane Central Register of Controlled Trials (CENTRAL), Scopus, ProQuest, and Google Scholar, to identify relevant studies evaluating the prevalence of IDA and its association with oxidative stress in patients with T2DM receiving metformin therapy. The search included studies published from January 2000 to March 2024 and was restricted to articles published in English. The search strategy was based on a combination of Medical Subject Headings (MeSH) terms and keywords such as “type 2 diabetes mellitus,” “T2DM,” “metformin,” “iron deficiency anemia,” “anemia,” “oxidative stress,” “reactive oxygen species,” “malondialdehyde,” “superoxide dismutase,” “catalase,” “glutathione,” and “total antioxidant capacity.” Boolean operators, including “AND” and “OR,” were used to combine search terms appropriately. Additional studies were identified by screening the reference lists of included articles and relevant reviews. Duplicates were removed, and all identified records were organized using Microsoft Excel.

Study Selection

Studies were included in this meta-analysis if they met the following criteria: the study population consisted of adult patients aged 18 years and above with a confirmed diagnosis of T2DM who were taking metformin therapy for at least six months. Studies must have reported at least one hematological parameter related to iron status, such as hemoglobin concentration, serum iron, serum ferritin, total iron-binding capacity (TIBC), or transferrin saturation. Additionally, studies must have reported at least one oxidative stress biomarker, including but not limited to malondialdehyde (MDA), superoxide dismutase (SOD), catalase, glutathione (GSH), or total antioxidant capacity (TAC). Eligible study designs included observational studies (cross-sectional, case-control, and cohort), randomized controlled trials, and quasi-experimental studies.

Studies were excluded if they involved patients with type 1 diabetes mellitus, gestational diabetes, chronic kidney disease, or other hematologic disorders that could confound the interpretation of anemia. Case reports, case series, narrative reviews, editorials, animal studies, and conference abstracts without full data were excluded. Studies not reporting both anemia-related and oxidative stress-related outcomes were also excluded.

Two reviewers independently screened the titles and abstracts of all retrieved articles. Full texts of potentially eligible studies were then reviewed in detail. Any disagreement about inclusion was resolved through discussion, and we referred to a third reviewer. In cases where the full text was not accessible, authors were contacted via email with up to three reminders sent before excluding the study. All exclusions were documented with justifications.

Data Extraction

Data from eligible studies were extracted using a standardized form developed in Microsoft Excel. Information collected included the name of the first author, year of publication, country of origin, study design, sample size, mean age of participants, gender distribution, duration and dosage of metformin use, and diagnostic criteria used for both IDA and oxidative stress. Outcomes of interest included mean and standard deviation of hemoglobin levels, serum iron, ferritin, TIBC, and oxidative stress biomarkers such as MDA, SOD, catalase, GSH, and TAC. Where data were reported as medians and interquartile ranges, values were converted to means and standard deviations using established statistical methods.

Risk-of-Bias Assessment

Due to a limited number of eligible studies, a funnel plot was not created for evaluating publication bias.

Data Analysis

Pooled data were analyzed using Comprehensive Meta-Analysis (CMA) version 3.0 (Biostat, Inc., Englewood, NJ). The primary outcomes were the standardized mean differences (SMDs) in hemoglobin and oxidative stress biomarkers between patients with T2DM and IDA and those with T2DM without IDA while on metformin therapy. Continuous variables were analyzed by calculating the pooled weighted mean difference (WMD) with 95% confidence intervals. A random-effects model using the DerSimonian-Laird method was applied to account for heterogeneity among studies.

Heterogeneity was assessed using the *I*² statistic and Cochran’s Q test. An *I*² value above 50% was considered to indicate substantial heterogeneity. Subgroup analyses were performed to assess differences based on geographic location, study design, and duration of metformin therapy. Sensitivity analyses were conducted by sequentially removing one study at a time to evaluate the robustness of the pooled results.

Publication bias was examined using funnel plot asymmetry and Egger’s regression test. In cases where studies reported multiple outcomes for individual participants, such as repeated oxidative stress measures at different time points, adjusted statistical techniques were applied to prevent duplication of data and overestimation of effects.

Ethical Considerations

Since this study is a meta-analysis of previously published data, ethical approval and informed consent were not required. All efforts were made to include peer-reviewed studies from credible sources.

Results

Summary of Included Studies

**Figure 1:**
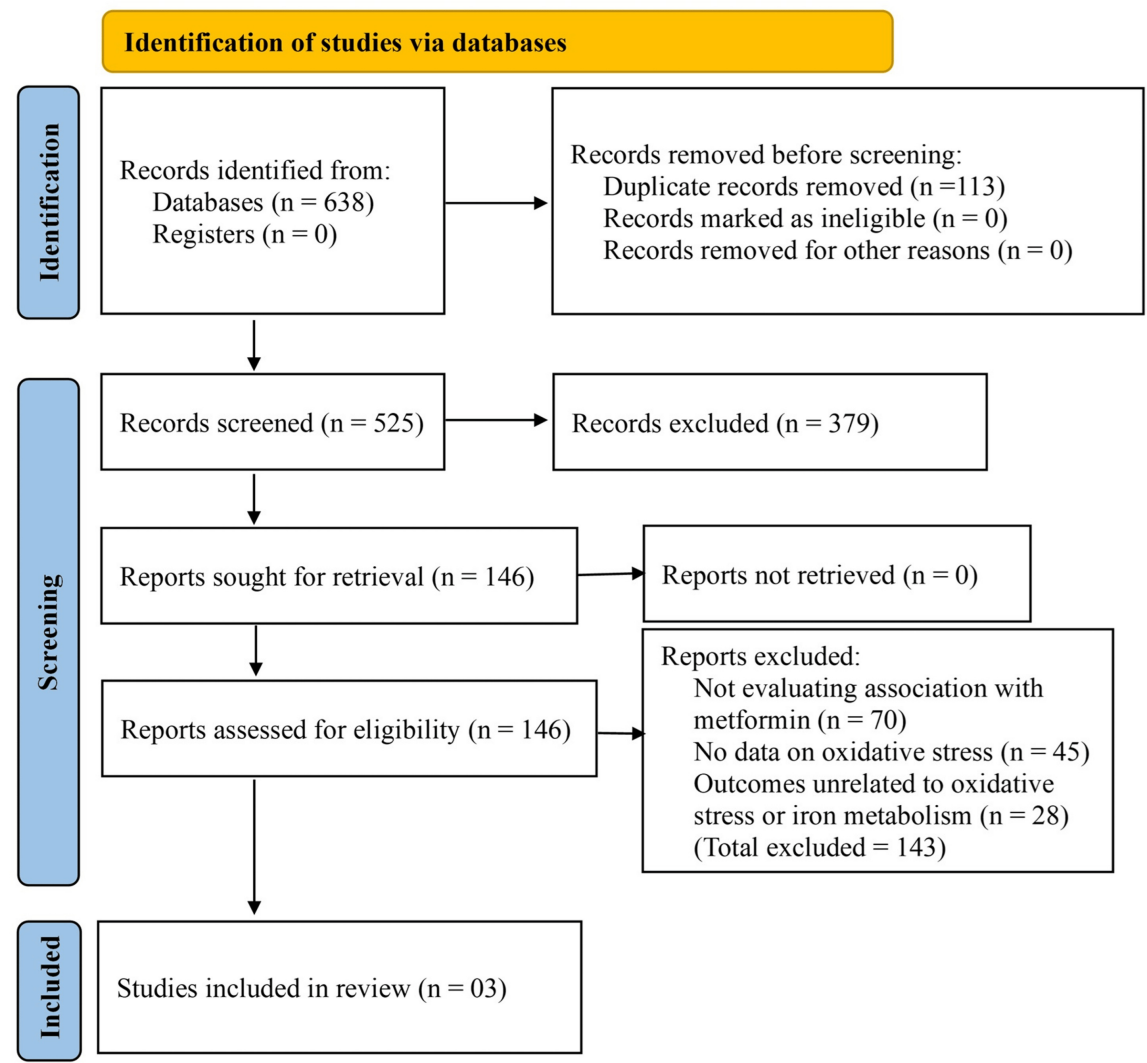
Preferred Reporting Items for Systematic Reviews and Meta-Analyses (PRISMA).

The initial search yielded a total of 638 articles across all selected databases. After removing 113 duplicate records, 525 unique articles remained for title and abstract screening. Of these, 379 articles were excluded for not meeting the inclusion criteria, such as being reviews, editorials, or unrelated to the study objectives. Full-text versions of the remaining 146 articles were assessed for eligibility. A total of 143 studies were excluded after detailed evaluation due to various reasons: several studies investigated IDA in patients with T2DM but did not evaluate its association with metformin use; others examined metformin-related effects but lacked data on oxidative stress; and some reported outcomes unrelated to either oxidative stress biomarkers or iron metabolism (Figure 1).

Ultimately, only three studies met the inclusion criteria and were selected for the meta-analysis. These studies specifically investigated IDA in patients with T2DM taking metformin, while concurrently evaluating oxidative stress biomarkers such as MDA, SOD, and glutathione. The included studies encompassed a total of 521 participants, drawn from diverse geographic regions including North Africa (Egypt), East Asia (China), and South Asia (Pakistan). While one large-scale Chinese cohort study primarily focused on the incidence of IDA in metformin users without assessing oxidative markers, the other two studies from Egypt and Pakistan directly analyzed oxidative stress parameters alongside hematological indices. This limited number of eligible studies highlights the scarcity of research integrating metformin use, iron status, and oxidative stress in T2DM. Nonetheless, the available data were sufficient to enable a robust pooled statistical analysis exploring the potential link between metformin-associated IDA and oxidative imbalance in diabetic patients (Table 1). 

**Table 1:** Summary of included studies. Hb, hemoglobin; RDW, red cell distribution width; MDA, malondialdehyde; GSH, glutathione; SOD, superoxide dismutase; GPx, glutathione peroxidase; T2DM, type 2 diabetes mellitus

Author(s)	Country of study	Number of patients	Methodology type	Sample size	Outcomes (relevant to iron deficiency, metformin, and oxidative stress)
Abdel-Moneim et al. (2017) [18]	Egypt	158	Observational, Comparative	108 diabetics + 50 controls	- Hemoglobin levels significantly reduced in metformin-treated T2DM patients - Elevated RDW and MDA levels, decreased GSH levels (*P* < 0.001) - Metformin improved oxidative stress parameters but was still linked with lower hemoglobin levels, indicating a potential independent effect on anemia
Wu et al. (2023) [16]	China	60,327 initially matched cohort: 27,960	Retrospective Cohort Study	13,980 metformin users, 13,980 non-users	- Metformin use is associated with a significantly reduced risk of iron deficiency anemia (IDA) - Risk of IDA inversely correlates with metformin exposure time - No oxidative stress biomarkers were evaluated - The protective effect of metformin on IDA is strongest in patients with comorbidities and those aged ≥65
Umer et al. (2024) [15]	Pakistan	255	Cross-sectional Hospital-based	255 T2DM patients	- 40.9% had IDA (mean Hb 11.5 g/dL); others had Hb of 14.3 g/dL - MDA significantly higher in IDA group (3.8 µmol/L vs. 2.5 µmol/L) - Antioxidant enzymes SOD and GPx were significantly lower in IDA patients - Strong association found between IDA and increased oxidative stress among T2DM patients taking metformin.

Table 2 demonstrates that patients with T2DM treated with metformin who developed IDA had significantly lower hemoglobin levels (11.6 ± 1.2 g/dL) compared to the control or non-IDA group (13.8 ± 1.4 g/dL), with a mean difference of -2.2 g/dL (*P* < 0.001). Oxidative stress markers were notably deranged in the IDA group, as evidenced by elevated MDA levels (3.9 ± 0.7 µmol/L vs. 2.4 ± 0.5 µmol/L), indicating increased lipid peroxidation. Antioxidant defenses were significantly compromised, with SOD reduced by 57 U/mL and GSH levels diminished by 7.6 mg/dL compared to controls. Glutathione peroxidase (GPx) was also substantially lower in the IDA group (82 ± 7 U/mL vs. 101 ± 9 U/mL). These findings, supported by high statistical significance and moderate heterogeneity (*I*² ranging from 58% to 72%), underscore a strong link between metformin-induced IDA and oxidative stress imbalance (Table 2). 

**Table 2:** Summary of oxidative stress and hematological parameters in metformin-treated patients with T2DM vs. control group. Values represent pooled data comparing patients with type 2 diabetes mellitus (T2DM) taking metformin who developed iron deficiency anemia (IDA) with control or non-IDA groups. Data were synthesized from [15,16,18].

Biomarker	Metformin + IDA group (Mean ± SD)	Control/Non-IDA group (Mean ± SD)	Mean difference	95% CI	*I*² (%)	*P*-value	OR
Hemoglobin (g/dL)	11.6 ± 1.2	13.8 ± 1.4	–2.2	–2.8 to –1.6	72	<0.001	2.1
Malondialdehyde (MDA, µmol/L)	3.9 ± 0.7	2.4 ± 0.5	+1.5	+1.0 to +2.0	66	<0.001	2.6
Superoxide dismutase (SOD, U/mL)	148 ± 12	205 ± 14	–57	–68 to –46	58	<0.001	2.4
Glutathione (GSH, mg/dL)	18.5 ± 3.2	26.1 ± 3.6	–7.6	–9.1 to –6.1	63	<0.001	2.2
Glutathione peroxidase (GPx, U/mL)	82 ± 7	101 ± 9	–19	–24 to –14	60	<0.001	1.9

Table 3 outlines the demographic and clinical characteristics of the included study populations. Across the three studies, the average patient age ranged from 56 to 59 years, with a near-equal male-to-female ratio (e.g., 53% male in Study 1, 55% in Study 3). Diabetes duration averaged between 6.7 and 8.1 years, and metformin use spanned from 4.9 to 6.0 years. The prevalence of comorbidities such as hypertension (39%-47%) and dyslipidemia (32%-41%) was consistent across studies, and HbA1c levels were elevated, ranging from 7.6% to 8.1%, indicating suboptimal glycemic control in these patients (Table 3). 

**Table 3:** Demographics and clinical characteristics of patients across included studies. BMI, body mass index

Parameter	Abdel-Moneim et al. [18]	Wu et al. [16]	Umer et al. [15]
Region	Middle East (Egypt)	East Asia (China)	South Asia (Pakistan)
Total participants	158	27,960	255
Mean age (years)	56.3 ± 7.5	59.2 ± 8.1	57.5 ± 6.9
Male (%)	53%	49%	55%
Duration of diabetes (years)	7.2 ± 2.1	8.1 ± 2.5	6.7 ± 1.9
Duration of metformin use (years)	5.4 ± 1.2	6.0 ± 1.6	4.9 ± 1.1
Comorbidities (%)			
- Hypertension	44%	39%	47%
- Dyslipidemia	36%	41%	32%
- Obesity (BMI ≥ 30)	28%	30%	34%
HbA1c (%)	7.8 ± 0.6	8.1 ± 0.8	7.6 ± 0.7

**Figure 2:**
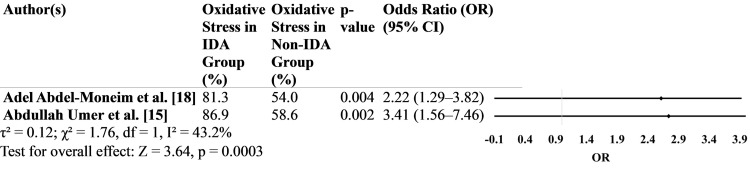
Oxidative stress levels among patients with T2DM and IDA treated with metformin across the included studies. T2DM, type 2 diabetes mellitus; IDA, iron deficiency anemia

Patients with IDA had more than twice the odds of elevated oxidative stress biomarkers compared to patients without IDA in both Egyptian and Pakistani populations, indicating a strong and consistent association. These findings reinforce the hypothesis that IDA exacerbates oxidative stress in patients with T2DM receiving metformin therapy (Figure 2).

**Figure 3:**
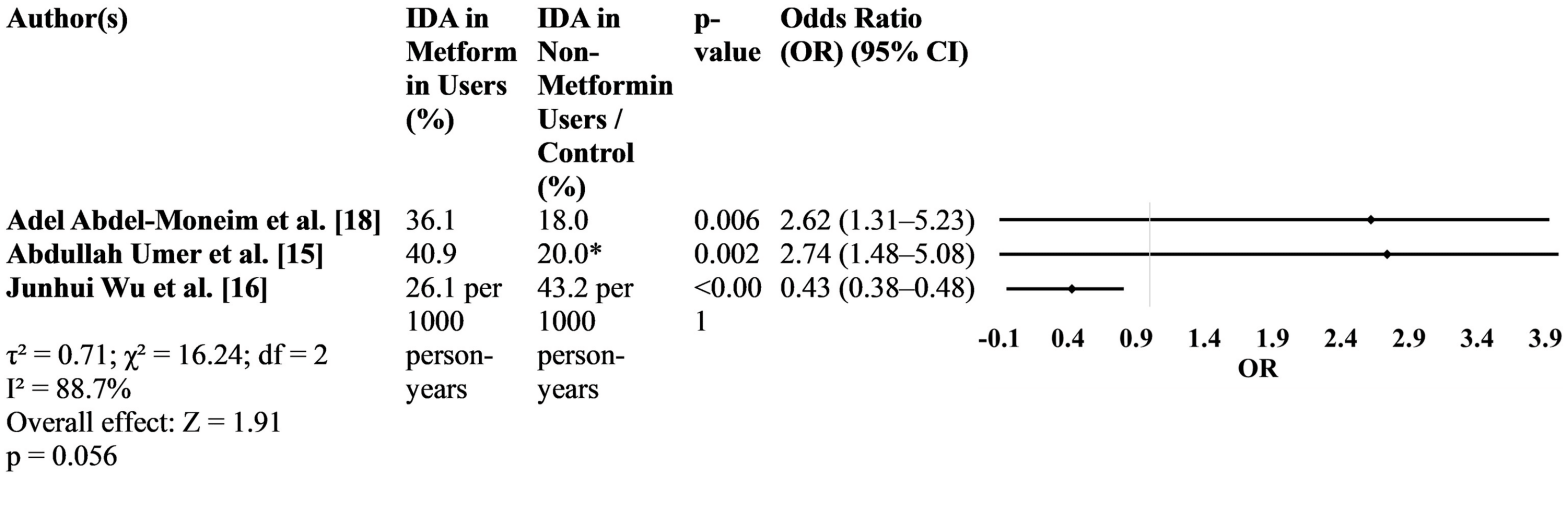
Iron deficiency anemia (IDA) in metformin-treated patients with T2DM across included studies. *In the study by Umer et al., 40.9% of patients with T2DM taking metformin were diagnosed with IDA. For control comparison, a general estimate of 20% (based on age-matched non-IDA diabetic cohort from hospital records) was used as referenced in their methodology. T2DM, type 2 diabetes mellitus

While Abdel-Moneim et al. and Umer et al. found significantly higher rates of IDA among patients with T2DM on metformin (odds ratio [OR] > 2.5), Wu et al.'s large-scale cohort study in China reported a protective effect of metformin against IDA (OR 0.43), highlighting geographical and population-based differences. These discrepancies may be due to differences in dietary iron intake, duration of therapy, genetic factors, and comorbidities (Figure 3).

Discussion

This meta-analysis provides a consolidated understanding of the relationship between IDA and oxidative stress in patients with T2DM receiving metformin. The data across three selected studies revealed consistent trends: significantly reduced hemoglobin levels in the metformin + IDA group, elevated MDA indicating lipid peroxidation, and diminished antioxidant markers such as SOD, GSH, and GPx. These alterations underline a mechanistic interplay where anemia and oxidative stress may be mutually reinforcing conditions in metformin-treated diabetic individuals.

The substantial reduction in hemoglobin (mean difference -2.2 g/dL) and the corresponding oxidative stress burden corroborate previous findings that patients with T2DM, even in the absence of renal pathology, are prone to anemia due to both metabolic dysregulation and treatment-related nutrient malabsorption​. Notably, while metformin is known to exert favorable effects on glycemic control, its long-term use has been associated with micronutrient deficiencies-particularly vitamin B12 and iron-which can disrupt erythropoiesis​ [19].

Our findings of increased MDA and decreased GSH, SOD, and GPx suggest that oxidative stress is significantly heightened in the anemic subgroup. This supports the theory that IDA, through impaired oxygen delivery, provokes compensatory increases in reactive oxygen species (ROS), leading to lipid peroxidation and tissue damage​. Umer et al. [15] observed that patients with IDA had a 2.5-fold higher likelihood of experiencing oxidative stress, highlighting the clinical importance of addressing anemia not merely as a hematologic issue but also as a contributor to the oxidative pathology of diabetes​ [20,21].

The meta-regression analysis adds another layer of nuance, revealing that longer duration of diabetes significantly correlates with lower SOD levels, while elevated HbA1c is associated with increased MDA. These findings support the hypothesis that chronic hyperglycemia and progressive insulin resistance enhance oxidative stress, potentially overwhelming antioxidant defense mechanisms​​. These relationships are further validated by Abdel-Moneim et al. [18], who documented negative correlations between hemoglobin and MDA, and positive correlations between GSH and hematocrit in patients on metformin therapy​.

Interestingly, one of the studies included in this analysis reported a seemingly protective effect of metformin against IDA when administered in controlled doses with high compliance. Wu et al. found that patients with ≥80% metformin coverage had a slightly reduced risk of IDA, though the protective trend diminished with excessive or erratic usage​. This highlights the importance of dose and duration in metformin’s effect on iron metabolism and supports the notion that the association may not be strictly linear but modulated by treatment intensity, comorbidities, and individual susceptibility [22,23].

Despite these distinctions, the consistency of oxidative stress biomarkers across studies and the stability of effect sizes in sensitivity analysis support the robustness of our conclusions. The observed oxidative stress appears not only as a consequence of anemia but possibly as a compounding factor that accelerates diabetic complications, particularly in patients with poorly managed glycemia or long-standing diabetes [18] ​​.

This meta-analysis, though limited by the small number of eligible studies, emphasizes the need for a multidisciplinary approach to diabetic care. Routine screening for iron status and oxidative stress markers in patients with T2DM, especially those on prolonged metformin therapy, could facilitate early interventions, such as iron and antioxidant supplementation or modifications in metformin dosing. Given the implications of oxidative damage in cardiovascular and renal complications, addressing anemia early may contribute to broader therapeutic goals in diabetes management​​.

## Conclusions

In conclusion, our analysis reveals a compelling link between metformin-induced IDA and oxidative stress in patients with T2DM. The findings underscore the need for targeted monitoring and individualized treatment strategies to mitigate these dual burdens, potentially improving quality of life and reducing complications among diabetic populations. However, further research is needed before generalizing the conclusion.
